# Obituary—Dr. Geo. W. McElhaney

**Published:** 1893-11

**Authors:** 


### Obituary—Dr. Geo. W. McElhaney.

Died, in Columbus, Ga., on the morning of September 15th, after an illness of a few days, Dr. George W. McElhaney, in the fiftieth year of his age.

Dr. McElhaney was one of the older dentists of the south; he stood high in the esteem of his professional brethren; he was a man of large attainments, and devoted his best energies and efforts to the welfare of his chosen profession. He was highly esteemed by all who knew him; a man of generous nature, and attractive in manner. His death will make a breach in the profession, and especially in that of the south, that will not be readily filled.

His bereaved and sorrowing family will have the sincere sympathy of all his friends.

In corroboration of what is here said, the following preamble and resolutions passed by the dentists of Columbus, Ga., are here given:

"*Whereas*, The grim monster Death has invaded the ranks of our profession and removed from our midst a conspicuous and honored member, Dr. George W. McElhaney, it is appropriate that some public expression should be given of our keen regret at the loss we have sustained, therefore be it

"*Resolved*, That in the death of Dr. George W. McElhaney the dental profession of Columbus has been deprived of a valued and enthusiastic member, a generous hearted and affable friend, and the community of a useful and honorable citizen.

His extraordinary ability and skill as an operator placed him among the foremost dentists of the South. His zeal and love for his profession were recognized in his work in promoting its highest interests, and his unswerving duty as a member of the Georgia State and Southern Dental Societies will ever be remembered by his associates.

"*Resolved*, That our sincerest sympathies are tendered his bereaved wife in her deep affliction.

"*Resolved*, That this simple tribute to his worth as a man, and his fame as a dentist be published in the daily papers as expressive of our sentiments.

"C. T. Osborn,
"W. T. Pool,
"Aug. Burghard,
"*Committee.*"

